# Multiple tail vein injections of adipose-derived mesenchymal stem cells ameliorate allergic rhinitis in mice: superior efficacy of prolonged regimens

**DOI:** 10.3389/fimmu.2025.1678927

**Published:** 2025-11-25

**Authors:** Yutong Xie, Jiacheng Zhang, Wenhan Yang, Zhiyu Pan, Lian Wang, Ju Lai, Kai Fan, Yalei Dai, Keqiang Zuo, Fei He, Zhengliang Gao, Shaoqing Yu

**Affiliations:** 1 Department of Otolaryngology, Head and Neck Surgery, Tongji Hospital, School of Medicine, Tongji University, Shanghai, China; 2 Department of Cardiac and Vascular Surgery, Huaihe Hospital of Henan University, Kaifeng, Henan, China; 3 China-Japan Friendship Medical Research Institute, Shanghai University, Shanghai, China; 4 Department of Interventional and Vascular Surgery, Affiliated Hospital of Jinggangshan University, Ji’an, Jiangxi, China; 5 Department of Allergy, Tongji Hospital, School of Medicine, Tongji University, Shanghai, China; 6 Kaifeng City Key Laboratory of Vascular Diseases, Kaifeng, China

**Keywords:** allergic rhinitis, adipose-derived mesenchymal stem cells, tail vein injection, ovalbumin-induced murine, immunomodulation, therapeutic efficacy

## Abstract

**Background:**

Adipose derived mesenchymal stem cells (ADSCs) are a subset of mesenchymal stem cells (MSCs), showing broad anti allergic effects in type 2 inflammation. Their systemic efficacy in allergic rhinitis (AR) is not well defined. In this study, we tested whether prolonged and repeated ADSC delivery improves outcomes in the AR mouse model.

**Methods:**

An ovalbumin (OVA) induced AR mouse model was established. Mice were divided into three groups: a control group, an AR model group, and an ADSC treatment group. Each group was administered phosphate buffered saline (PBS) or ADSCs via tail vein infusion during defined treatment phases. Symptom severity including nasal scratching and sneezing was recorded before and after treatment. Nasal mucosal pathology and inflammatory biomarkers were assessed at the same time points. Therapeutic efficacy was evaluated by the therapeutic efficacy index (TEI).

**Results:**

Systemic tail vein injection of ADSCs significantly attenuated AR symptoms and nasal inflammation. Treated mice exhibited decreased frequencies of nasal scratching and sneezing. Consistently, serum specific immunoglobulin E (sIgE), immunoglobulin G1 (IgG1) and transforming growth factor beta (TGF-β) were also reduced. Concurrently, both transcriptional and cytokine profiling indicated an increased ratio of T helper 1 (Th1) to T helper 2 (Th2) related cytokines, indicating restoration of immune balance. Besides, long-term (4-weeks) ADSC therapy with multi-injection yielded superior efficacy over short-term (1-week and 2-weeks) regimens in therapeutic efficacy index (TEI) analysis.

**Conclusion:**

Systemic ADSC delivery through the tail vein alleviated AR in mice. Extended multi-injection schedules produced greater benefit. ADSCs represent a promising systemic immunomodulatory therapy for AR, with enhanced efficacy under longer treatment cycles.

## 1 Introduction

Allergic rhinitis (AR) is a chronic nasal mucosal inflammation disorder induced by allergen exposure. Recent epidemiology shows an increasing global burden, with marked impact on children and adolescents (1, 2). The typical clinical manifestations of AR include nasal itching, sneezing, rhinorrhea, and nasal congestion (3). Notably, severe AR may progress to comorbid allergic conjunctivitis and/or asthma (4, 5). AR is an immunoglobulin E (IgE)-mediated disease driven by T helper 2 (Th2) immune response. AR induces Th2 differentiation and robust secretion of interleukin 4 (IL-4), interleukin 5 (IL-5), and interleukin 13 (IL-13), promoting IgE class switching, eosinophilic inflammation, and mucus hypersecretion (6). Suppression of T helper 1 (Th1) cytokines and impairment of regulatory T-cell (Treg) function further reinforce Th2 predominance. This maintains the Th1/Th2 imbalance and a chronic inflammatory milieu in the nasal mucosa (7, 8). Recent studies show that the T helper 17 (Th17) and Treg imbalance also contributes to persistent inflammation in AR. Elevated Th17 activity and reduced Treg function promote interleukin 17 (IL-17) mediated inflammation and mucosal damage (9, 10). Current treatments for AR including antihistamines, corticosteroids, and immunotherapy, provide limited efficacy (11), underscoring the need for novel therapeutic strategies.

Adipose-derived mesenchymal stem cells (ADSCs) are a stromal subset isolated from adipose tissue. These cells are characterized by the expression of specific surface markers cluster of differentiation 73 (CD73), cluster of differentiation 90 (CD90), and cluster of differentiation 105 (CD105), while being negative for cluster of differentiation 45 (CD45), cluster of differentiation 14 (CD14), cluster of differentiation 34 (CD34), and human leukocyte antigen DR isotype (HLA-DR) (12, 13). ADSCs exhibit typical plastic-adherence, expansion capacity *in vitro*, and multipotent differentiation potential into osteogenic, chondrogenic, adipogenic, and myogenic lineages (13). Compared to mesenchymal stem cells (MSCs) from bone marrow, umbilical cord, or other tissues, harvesting of ADSCs is more convenient and minimally invasive. Larger tissue volumes can be obtained from adipose sources, resulting in a greater initial cell yield (14). Importantly, ADSCs exert immunomodulatory effects by sensing the inflammatory microenvironment and releasing a diverse range of secreted factors. They can release both pro-inflammatory mediators such as tumor necrosis factor alpha (TNF-α), interleukin 1 beta (IL-1β) (15), and interleukin 8 (IL-8) (16), and anti-inflammatory signals such as interleukin 10 (IL-10), interleukin 4 (IL-4), and interleukin 13 (IL-13) (17) to modulate immune responses. ADSCs also exert immunoregulatory effects through extracellular vesicles and exosomes that carry bioactive molecules (18). Through soluble factors and direct cell–cell interactions, adipose-derived stem cells can modulate the activity and function of T cells, B cells, macrophages, dendritic cells, and natural killer cells (18). The immunoregulatory and tissue repair capacities of ADSCs have been validated in multiple disease models, including asthma (19), rheumatoid arthritis (20), inflammatory bowel disease (21), and graft-versus-host disease (22, 23). In allergic conditions, ADSCs promote the induction and expansion of Tregs, which in turn suppress effector T cell responses (24). Besides, extracellular vesicles derived from ADSCs inhibit the differentiation and effector functions of Th2 cells, thereby attenuating the type 2 cytokine milieu (25). Through these combined effects, ADSCs help rebalance the Th1/Th2 axis, restoring immune homeostasis (26).

Most studies have focused on mesenchymal stem cells (MSCs) derived from bone marrow, umbilical cord, or other tissues in animal models of allergic rhinitis, reporting reduced symptoms, decreased IgE, IL-4, IL-5, and IL-10, and increased interferon gamma (IFN-γ) following treatment (27–30). However, studies on adipose derived mesenchymal stem cells (ADSCs), a clinically attractive source of MSCs, remain limited. Our previous study showed that extracellular vesicles derived from adipose derived mesenchymal stem cells (ADSC-EVs) alleviated AR, lowering symptoms, sIgE and Th2 cytokines, and improving nasal tissue pathology (26). In another study of ours, we compared tail-vein, intranasal, and combined administration of ADSC-EVs in AR mice to define delivery strategy (31). Recent work further confirms that ADSC-EVs improve AR symptoms, decrease inflammatory factor levels, and reduce goblet cells and eosinophils (32). However, studies employing ADSC cells, rather than ADSC-EVs, are relatively scarce, even though cell infusion may confer distinct advantages such as homing to inflamed tissues and sustained paracrine immunomodulation (31, 33, 34). Moreover, the impact of treatment cycles on efficacy has not been systematically evaluated. Our previous study using ADSC-EVs evaluated repeated dosing schedules (26), whereas similar optimization for intravenous cell infusion has not yet been reported.

In this study, we directly administered ADSCs via tail vein injection to evaluate their therapeutic potential in AR mice, with a focus on comparing the efficacy of short term and prolonged multi-injection regimens. We systematically compared nasal histopathology, profiled Th1/Th2 cytokines, and assessed immune cell frequencies to determine whether extended treatment schedules provide additional therapeutic benefit. By incorporating a therapeutic efficacy index (TEI), this study aims to guide rational treatment regimen design and facilitate the clinical translation of ADSC-based therapies for AR.

## 2 Materials and methods

### 2.1 Mice

27 healthy male BALB/c mice aged 6–8 weeks were used. Body weight was approximately 20-30g. Animals were housed under specific pathogen free conditions in the animal experiment center of Tongji hospital. After 1-week acclimatization period, the mice were randomly assigned to three main groups: the CON group (9 mice), the AR group (9 mice), and the ADSC group (9 mice). Each group was further divided into three subgroups, corresponding to 1-week, 2-week, and 4-week treatment durations, with 3 mice in each subgroup.

### 2.2 Induction of AR in mice

**Figure 1:**
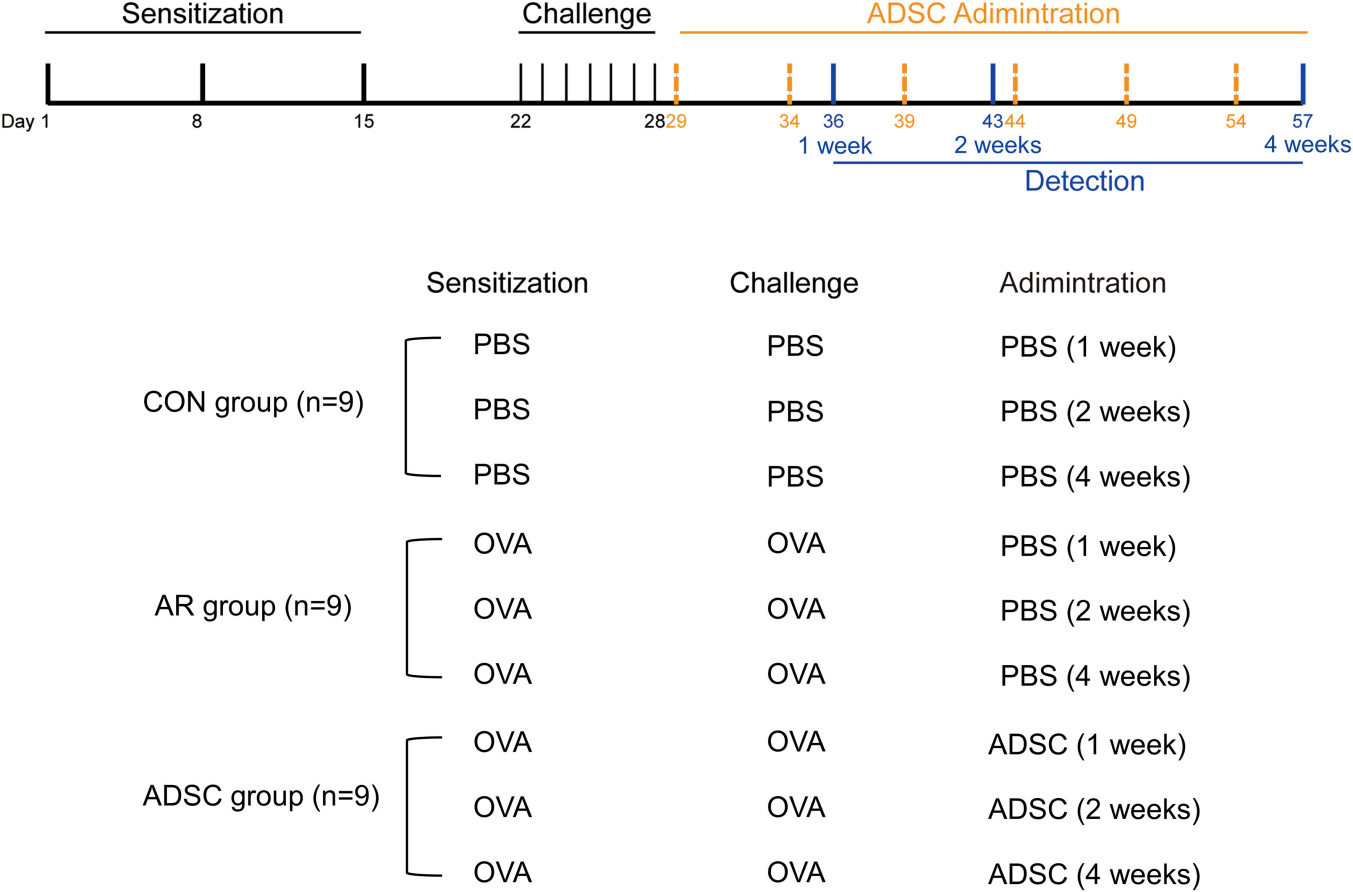
Experimental timeline. Sensitization was performed on days 1, 8, and 15 using intraperitoneal injections of OVA emulsified with aluminum hydroxide. Mice were challenged with intranasal instillation of OVA once daily on days 22–28. ADSCs were delivered via tail vein on days 29, 34, 39, 44, 49, and 54. All groups were terminated on day 36 (1 week), day 43 (2 weeks), or day 57 (4 weeks) for endpoint assessments. CON group (n = 9): PBS was used during sensitization, challenge, and administration. AR group (n = 9): OVA was used for sensitization and challenge, with PBS during the administration phase. ADSC group (n=9): OVA was used for sensitization and challenge; ADSCs were given during the administration phase. Each group was further divided into three subgroups, corresponding to 1-week, 2-week, and 4-week treatment durations, with 3 mice in each subgroup.

AR mouse models were established as shown in **Figure 1**. On days 1, 8, and 15, each mouse was sensitized by intraperitoneal injection of 0.2 mg ovalbumin (OVA) mixed with 20 mg aluminum hydroxide powder. Mice in the control group received the same volume of sterile normal saline at the same intervals. Allergen challenge started on day 22 after sensitization. A solution containing 40 mg OVA in 1 mL sterile normal saline was prepared for this procedure. AR mice were challenged intranasally with 10 µL of OVA solution per nostril once daily for seven consecutive days. Control mice received the same volume of sterile normal saline prepared and administered under identical conditions.

### 2.3 Administration of ADSCs

Human adipose-derived mesenchymal stem cells (hADSCs) were obtained from the Stem Cell Laboratory of Tongji University, provided by Dr Shi and Dr Wang. ADSCs were isolated from human adipose tissue by collagenase digestion and expanded by adherent culture. The ADSC phenotype had been verified in the earlier studies from Dr Shi (35–37). Flow cytometry has been used to immunophenotype the ADSCs in our prior study (26). Cells were positive for CD73 and CD90 and negative for CD34, CD45, HLA-DR, CD116, and CD9 (26).

ADSCs from the 3rd to 5th passages were utilized. Before administration, cells were centrifuged at 1300 revolutions per minute (rpm) for 3 minutes and resuspended in phosphate-buffered saline (PBS) to prepare a cell suspension. Mice in the ADSC administration group received 1.5×10^6^ cells per mouse every five days from day 29. Mice in the control and AR groups were injected with an equal volume of PBS at the same time points. Behavioral and experimental assessments were performed 1, 2, and 4 weeks after successful establishment of the AR model (**Figure 1**).

### 2.4 Symptom evaluation

Mice were euthanized at three time points, namely 1, 2, and 4 weeks after administration. Before each euthanasia, nasal symptoms were observed and recorded for 15 minutes following stimulation with the same allergen solution. Symptom severity was evaluated according to the established scoring criteria showed in **Table 1**.

**Table 1:** General symptomatology score of mice.

Score	Rubbing	Sneezing	Clear nasal discharge
1	Scratching with one paw several times	1~3	Clear nasal discharge flows to the anterior nostrils
2	Scratching several times with both claws	4~10	Clear nasal discharge extends beyond the anterior nostrils
3	Rubing everywhere	>11	Tears streaming down one’s face

### 2.5 Flow cytometry

Flow cytometry was performed to evaluate Th1 and Th2 cell levels in mouse spleens. After euthanasia, spleen tissues were carefully dissected and collected. The spleens were mechanically dissociated, and the resulting cell suspensions were centrifuged to obtain lymphocytes. Lymphocytes were stained with fluorescein isothiocyanate (FITC)-conjugated anti-mouse CD4, allophycocyanin (APC)-conjugated anti-mouse IL-4, and Brilliant Violet 421 (BV421)-conjugated anti-mouse IFN-γ antibodies, following the manufacturer’s protocol of the staining kit. The stained cells were analyzed using a flow cytometer to determine the proportions of Th1 (CD4^+^ IFN-γ^+^) and Th2 (CD4^+^ IL-4^+^) cells.

### 2.6 Hematoxylin and eosin staining and periodic acid–Schiff assay

After euthanasia, nasal mucosa tissues were carefully dissected and collected from the mice. The mucosa in the respiratory region was separated and fixed in 4% paraformaldehyde. The fixed tissues were dehydrated, cleared, and embedded in paraffin. Paraffin sections were dewaxed with graded xylene and alcohol solutions. H&E staining was performed by staining with hematoxylin for 5 minutes, rinsing under running water for 5 to 10 minutes, differentiating in 1% hydrochloric acid ethanol for 30 seconds, and counterstaining with eosin for 1 to 3 minutes. The sections were then dehydrated through graded ethanol and xylene, mounted, and observed under a microscope. For the PAS assay, nasal mucosa sections were oxidized with periodic acid and stained with Schiff’s reagent for 10 minutes, followed by hematoxylin staining for 3 minutes. The distribution and abundance of goblet cells were examined microscopically.

### 2.7 Enzyme-linked immunosorbent assay detection

Mice were euthanized by cervical dislocation, and peripheral blood was collected by enucleating the eyeballs. The concentrations of sIgE, IgG1, IL-4, IFN-γ, and TGF-β in peripheral blood were determined using ELISA kits, following the manufacturer’s instructions. Briefly, standard and sample solutions were added to pre-coated 96-well plates and incubated at 37°C for 1 hour. After washing, horseradish peroxidase (HRP)-conjugated secondary antibodies were added and incubated at 37°C for 30 minutes. The plates were washed again, and the substrate solution was added for color development. The reaction was terminated with stop solution, and absorbance was measured at 450 nm using a microplate reader.

### 2.8 Quantitative real-time polymerase chain reaction

Spleen tissues from mice were homogenized, and total RNA was extracted using the TRIzol reagent. The quality and concentration of RNA were assessed before further analysis. Complementary DNA (cDNA) was synthesized by reverse transcription according to the instructions provided with the reverse transcription kit. QPCR was then performed using a commercial qPCR kit to determine the mRNA expression levels of IL-4, IFN-γ, and forkhead box P3 (Foxp3). Each reaction was carried out in a 20 μL system containing cDNA, SYBR Green Master Mix, and gene-specific primers. The amplification conditions were as follows: initial denaturation at 95°C for 30 seconds, followed by 40 cycles of denaturation at 95°C for 5 seconds and annealing/extension at 60°C for 30 seconds. Melting curve analysis was performed to confirm amplification specificity. β-actin was used as the internal reference gene, and IL-4, IFN-γ, and Foxp3 were used as the target genes. The specific primer sequences were as follows:

β-actin:
5’-GGCTGTATTCCCCTCCATCG-3’, 5’-CCAGTTGGTAACAATGCCATGT-3’;
IL-4:
5’-GGTCTCAACCCCCAGCTAGT-3’, 5’-GCCGATGATCTCTCTCAAGTGAT-3’;
IFN-γ:
5’-ATGAACGCTACACACTGCATC-3’,5’-CCATCCTTTTGCCAGTTCCTC-3’;
Foxp3:
5’-CCCATCCCCAGGAGTCTTG-3’,5’-ACCATGACTAGGGGCACTGTA-3’

### 2.9 Therapeutic efficacy index analysis

The TEI was calculated to quantify treatment efficacy, defined as:


TEI = (Value_AR − Value_Treatment) / (Value_AR − Value_Control)

Value__AR_ = Measured value in the AR group

Value__Treatment_ = Measured value in the treatment group

Value__Control_ = Measured value in the control group

This metric reflects the proportion of the disease-induced pathology reversed by ADSC therapy, with TEI = 1 indicating complete restoration to the healthy state and TEI = 0 denoting no therapeutic effect.

### 2.10 Statistical analysis

All data are presented as mean ± standard deviation (SD). Statistical comparisons were performed using unpaired two-tailed Student’s *t*-tests. Specifically, comparisons were made between the CON group and the AR group, and between the AR group and the ADSC group at each timepoint. *P* < 0.05 was considered statistically significant. Analyses were performed in IBM SPSS Statistics (version 31.0.0; IBM Corp.) and GraphPad Prism (version 10.6.1; San Diego, CA, USA).

## 3 Results

### 3.1 Differentiation potential and nasal-targeted homing of ADSCs

**Figure 2:**
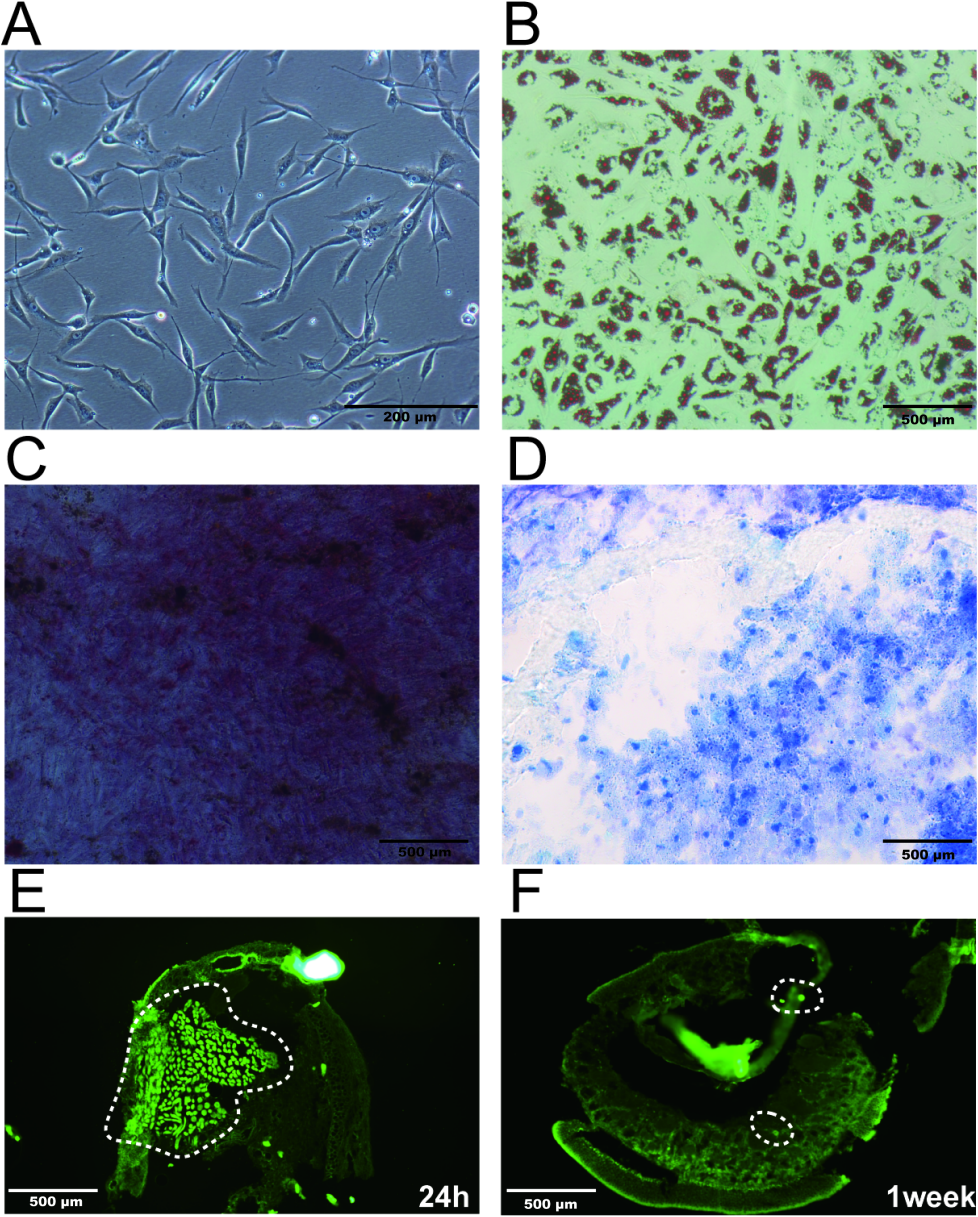
Characterization and nasal mucosal homing of ADSCs. **(A)** Representative morphology of ADSCs. **(B–D)** Multipotent differentiation of ADSCs: adipogenesis identified by Oil Red O staining **(B)**; osteogenesis by Alizarin Red staining **(C)**; chondrogenesis by Trypan Blue staining **(D)**. **(E)** Nasal mucosa 24 hours after tail-vein injection of fluorescently labeled ADSCs. **(F)** Nasal mucosa 1 week after tail-vein injection of fluorescently labeled ADSCs.

Consistent with their expected phenotype, human ADSCs provided by Dr. Shi and Dr. Wang exhibited a spindle-shaped morphology on microscopy (**Figure 2A**). These cells demonstrated marked multilineage differentiation potential, undergoing efficient adipogenic, osteogenic, and chondrogenic differentiation when induced with tri-lineage differentiation media (**Figures 2B–D**).

Histological examination of nasal mucosa sections confirmed efficient migration of intravenously delivered ADSCs to the nasal submucosa within 24 hours (**Figure 2E**). Critically, detectable ADSC populations persisted within nasal submucosal tissues for 7 days after injection, demonstrating their prolonged retention at the target site (**Figure 2F**).

### 3.2 ADSCs ameliorated symptoms and nasal mucosal pathology in AR mice

**Figure 3:**
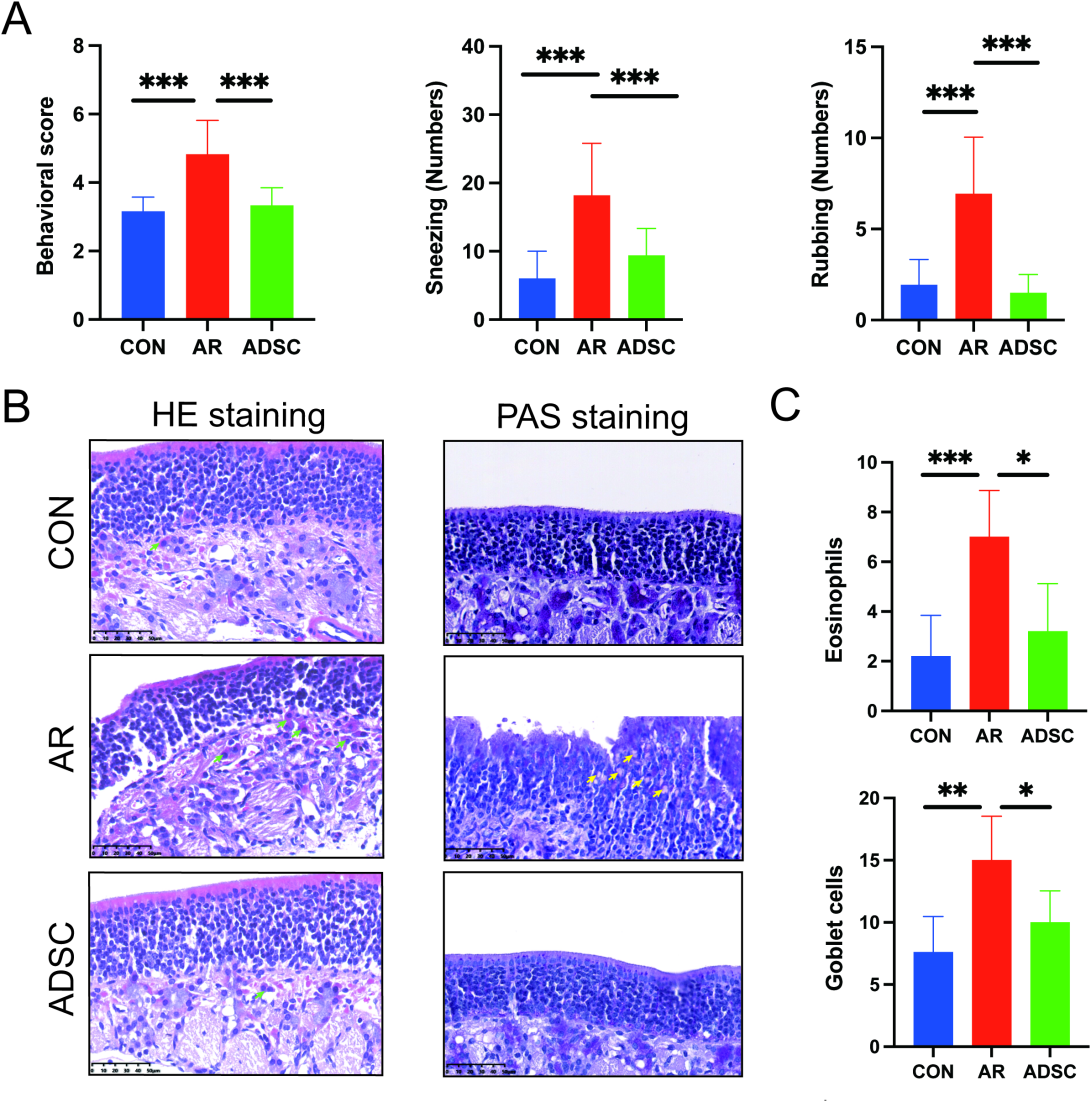
ADSC treatment attenuates allergic rhinitis symptoms and nasal mucosal pathology in OVA-induced AR mice. **(A)** Symptom scores, sneezing and nasal rubbing numbers (n = 6). **(B)** Representative histology of nasal mucosa: hematoxylin–eosin (H&E) and periodic acid–Schiff (PAS) staining (n = 9). Eosinophils are indicated by green arrows, and goblet cells are indicated by yellow arrows. **(C)** Quantification of eosinophils (from H&E; left panel in B) and goblet cells (from PAS; right panel in B) (n = 9). Data are shown as mean ± SD. *P < 0.05; **P < 0.01; ***P < 0.001.

To evaluate the AR mouse model, we systematically scored the behavioral symptoms of the mice, including the frequency of sneezing and nasal rubbing. Total symptom scores were calculated according to the detailed criteria outlined in **Table 1**. A cumulative score exceeding 5 points indicated successful model establishment. Both AR and ADSC groups exhibited significantly increased total symptom scores during the modeling (**Figure 3A**). Most mice demonstrated scores exceeding 5 points, displaying persistent nasal scratching with both forepaws, frequent sneezing episodes, paroxysmal neck scratching, nose-rubbing behavior against surfaces, and occasional lethargy. In contrast, control mice maintained consistently low symptom scores, confirming the successful establishment of the AR mouse model. Following ADSCs treatment, mice showed significantly reduced symptoms compared to the AR group, with pronounced decreases in sneezing frequency and nasal rubbing behaviors (**Figure 3A**).

Given the nasal tissue localization of AR symptoms, we examined histopathological changes in murine nasal mucosa. Hematoxylin and eosin (H&E) staining revealed intact and orderly nasal mucosa in control mice, with no evident pathological changes such as submucosal edema, mucosal rupture, or small vessel hyperplasia, and the eosinophil infiltration was minimal. In contrast, AR mice displayed a markedly thickened, disorganized epithelium with focal denudation and disordered cilia. Prominent eosinophil infiltration was present in the lamina propria (green arrows), with vasodilation and stromal loosening (**Figure 3B**). ADSC treatment significantly ameliorated these pathological alterations and reduced eosinophil infiltration (**Figures 3B, C**). Periodic acid–Schiff (PAS) staining demonstrated that the goblet cells were sparse in controls but markedly proliferated in AR mice (**Figure 3B and 3C**, yellow arrows). ADSC treatment significantly decreased goblet cell proliferation (**Figures 3B, C**). Collectively, ADSCs attenuated AR symptoms by suppressing eosinophil infiltration and reducing goblet cell hyperplasia in the nasal mucosa.

### 3.3 ADSCs suppressed allergic antibodies and restored Th1/Th2 balance in AR mice

**Figure 4:**
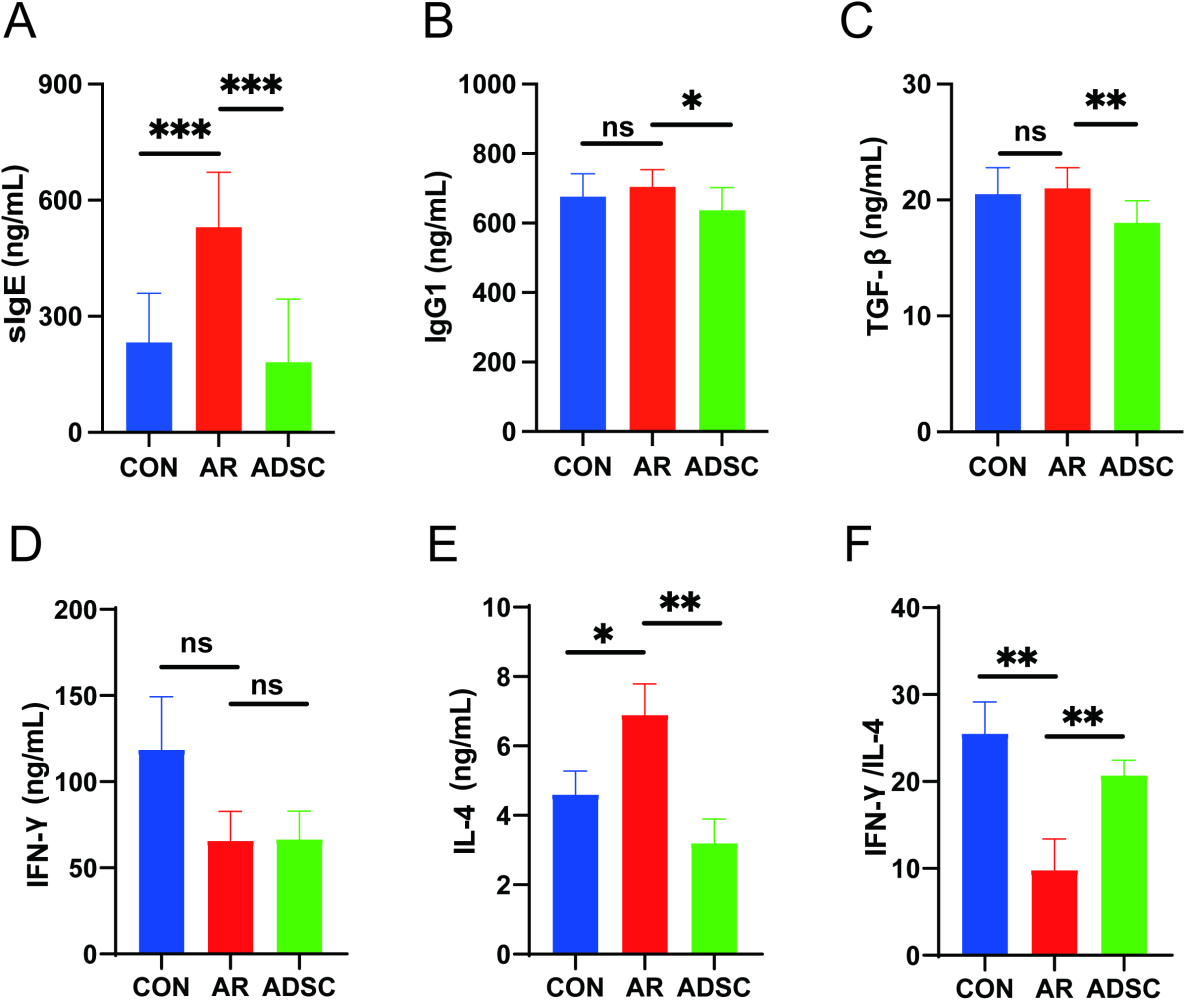
ADSCs attenuate allergic antibody production and rebalance Th1/Th2 cytokines. **(A–F)** Serum levels of sIgE **(A)**, IgG1 **(B)**, TGF-β **(C)**, IFN-γ **(D)**, IL-4 **(E)**, and IFN-γ/IL-4 ratio **(F)** measured by ELISA.Data are presented as mean ± SD. *P < 0.05; **P < 0.01; ***P < 0.001.

Elevated serum antigen-specific IgE is a hallmark sign of AR, while IgG1 contributes to immune responses during allergic reactions (38). Additionally, TGF-β exacerbates allergic symptoms by acting on mast cells and goblet cells during allergic attacks (39). In this study, ELISA demonstrated that OVA-stimulated AR mice exhibited significantly higher serum OVA-specific IgE levels (**Figure 4A**). ADSC treatment could markedly reduce these IgE levels, making their concentrations even slightly lower than those in the control group (**Figure 4A**). Similar patterns were observed for IgG1. While the AR group showed an upward trend in IgG1 secretion compared to controls, ADSC treatment significantly decreased IgG1 levels versus the AR group (**Figure 4B**). Furthermore, TGF-β was upregulated in AR mice and significantly decreased after ADSC treatment (**Figure 4C**). Collectively, these findings indicate that ADSCs alleviate allergic symptoms in AR mice by suppressing the secretion of key inflammatory mediators. Early research established that Th1/Th2 cell imbalance plays a pivotal role in initiating inflammation, particularly in the development and progression of allergic rhinitis (6, 26). Thus, we assessed Th1- and Th2-related cytokine profiles. IFN-γ (a key Th1 cytokine) secretion was reduced in the AR group and exhibited a subtle upward trend after ADSC treatment (**Figure 4D**). Conversely, IL-4 (a key Th2 cytokine) was significantly upregulated in the AR group, while ADSC administration significantly suppressed its secretion (**Figure 4E**). Critically, the IFN-γ/IL-4 ratio was significantly reduced in the AR group but significantly restored following ADSC treatment (**Figure 4F**), indicating a correction of Th1/Th2 skewing.

**Figure 5:**
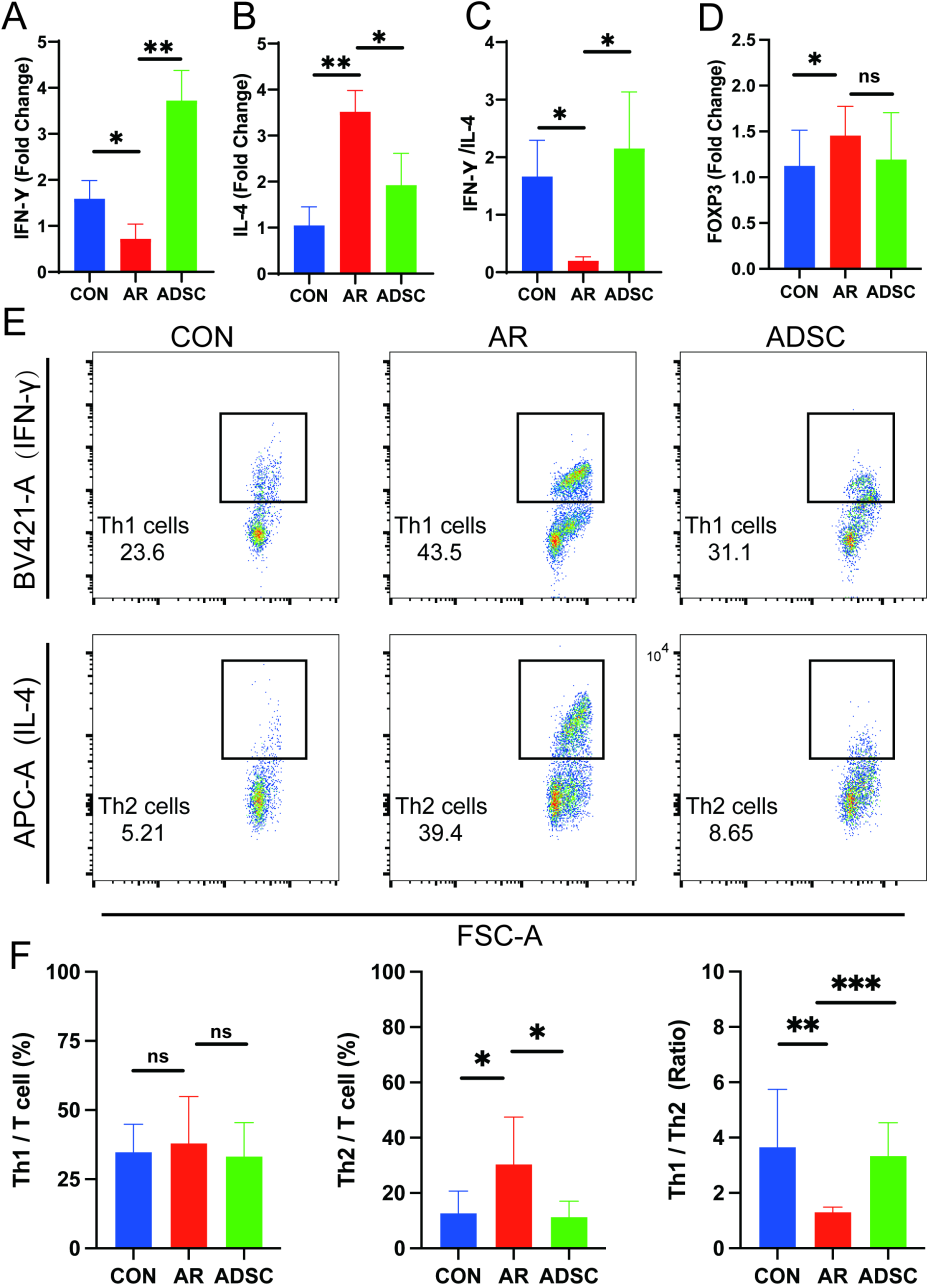
ADSC treatment rebalances systemic Th1/Th2 immunity in AR mice. (**A–D)** Splenic mRNA levels of IFN-γ **(A)**, IL-4 **(B)**, IFN-γ/IL-4 ratio **(C)** and FOXP3 **(D)** measured by qRT-PCR (n = 9). **(E)** Flow cytometry analysis of splenic Th1 and Th2 subsets (n=9). **(F)** Quantification of splenic Th1 and Th2 cells and the Th1/Th2 ratio derived from **(E)**. Data are expressed as mean ± SD. *P < 0.05; **P < 0.01; ***P < 0.001.

After quantifying cytokine secretion, we next examined the transcriptional levels of key regulators associated with Th1, Th2, and Treg responses. Quantitative real-time PCR (qRT-PCR) showed that IFN-γ transcripts were significantly reduced in the AR model group and increased following ADSC treatment (**Figure 5A**). Consistent with the ELISA results, IL-4 expression was markedly elevated in the AR group and decreased after ADSC treatment (**Figure 5B**). Critically, the IFN-γ/IL-4 expression ratio increased significantly after ADSC treatment (**Figure 5C**), in agreement with the earlier ELISA results (**Figure 4F**), supporting the restoration of Th1/Th2 balance. Moreover, qRT-PCR analysis revealed significant upregulation of FOXP3 (a key Treg marker) in the AR group, with a downward trend observed following ADSC treatment (**Figure 5D**). This elevation of FOXP3 may represent a compensatory regulatory response to allergic inflammation. Flow cytometry analysis further elucidated immune profiles among the three groups (**Figure 5E**). While Th1 cell proportions remained comparable between AR and ADSC groups, Th2 cells were significantly elevated in the AR group versus controls, and were significantly attenuated by ADSC treatment (**Figure 5F**). Critically, the Th1/Th2 ratio significantly increased in the ADSC-treated group compared to the AR group (**Figure 5F**). Collectively, these data demonstrated that AR exacerbates Th1/Th2 imbalance, while ADSC treatment effectively mitigates this dysregulation.

### 3.4 ADSCs ameliorate allergic rhinitis with duration-dependent immune modulation

**Figure 6:**
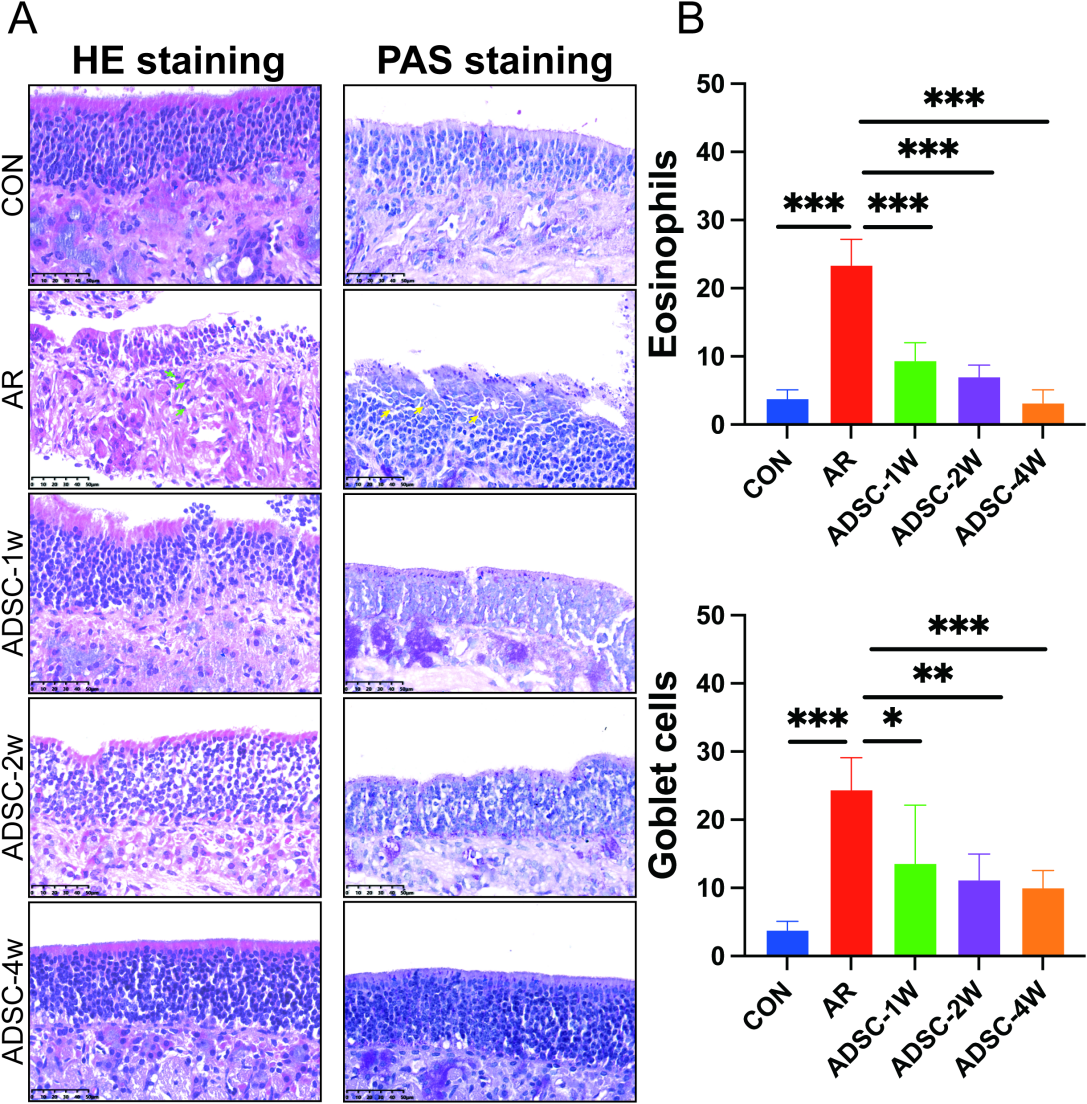
ADSC treatment attenuates nasal mucosal pathology across treatment durations. **(A)** Representative histology of nasal mucosa: hematoxylin–eosin (H&E) and periodic acid–Schiff (PAS) staining (n=9). Eosinophils are indicated by green arrows, and goblet cells are indicated by yellow arrows. **(B)** Quantification of eosinophils (from H&E; left panel in **B**) and goblet cells (from PAS; right panel in **B**) (n=9). Data are shown as mean ± SD. *P < 0.05; **P < 0.01; ***P < 0.001..

To optimize future ADSC therapeutic protocols, we further assessed the therapeutic efficacy of ADSCs varying treatment durations (1, 2, and 4 weeks), with each subgroup being compared separately to the AR group. Histopathological analysis of nasal tissue confirmed the therapeutic effects of ADSCs, demonstrating significantly reduced eosinophil infiltration and goblet cell proliferation across all treatment durations in AR mice (**Figures 6A, B**).

**Figure 7:**
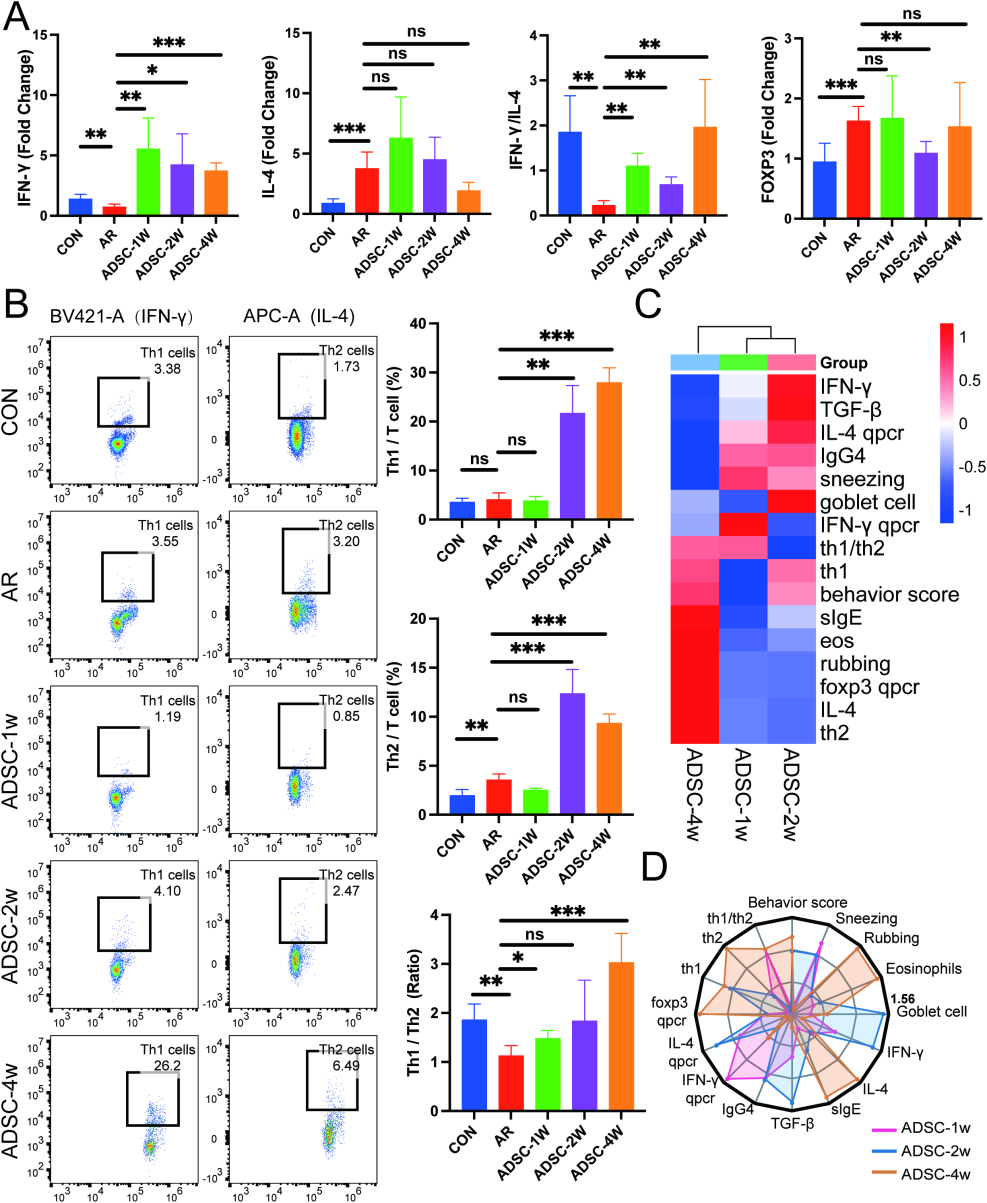
Four-week ADSC treatment achieves optimal Th1/Th2 balance and integrated therapeutic efficacy. **(A)** Splenic mRNA levels of IFN-γ, IL-4, IFN-γ/IL-4 ratio and FOXP3 measured by qRT-PCR (n = 9). **(B)** Flow cytometry analysis and quantification of splenic Th1 and Th2 subsets and the Th1/Th2 ratio (n=9). **(C)** Heatmap of TEI comparison across the three administration strategies. **(D)** Radar chart of TEI comparison across the three administration strategies. Data are expressed as mean ± SD. *P < 0.05; **P < 0.01; ***P < 0.001.

To further investigate Th1/Th2 differentiation across different treatment durations, we quantified transcriptional levels of key regulators IFN-γ and IL-4. Results showed that all ADSC treatment durations significantly alleviated the IFN-γ transcript suppression in AR mice (**Figure 7A**). Nevertheless, for IL-4 in AR mice, the therapeutic effects of the three treatment cycles were not ideal. Specifically, 1-week and 2-week ADSC treatments even exacerbated the upregulation of IL-4 transcriptional levels, while the 4-week group showed a non-significant downward trend (**Figure 7A**). Notably, all treatment cycles collectively mitigated the suppressed IFN-γ/IL-4 transcriptional ratio in AR mice (**Figure 7A**). Additionally, the transcriptional level of FOXP3, which is closely related to Treg differentiation, was upregulated in the AR group (**Figure 7A**), indicating that additional investigations are required to elucidate Treg-associated mechanisms in allergic rhinitis. Flow cytometry analysis revealed an increase in both Th1 and Th2 cell proportions after 2-and 4-week ADSC treatments, potentially associated with the immunological stage of the mice. Combined with Th1/Th2 ratio analysis, all treatment durations ameliorated Th1/Th2 imbalance in AR mice, with 1- and 4-week treatments significantly improving this imbalance (**Figure 7B**).

To enable unbiased cross-duration comparisons, AR controls were stratified by time (1, 2, and 4 weeks) to provide time-matched baselines for the ADSC groups. Because the AR model exhibits time-dependent variation in baseline severity, direct comparison of raw readouts across durations is confounded. We therefore derived a Therapeutic Efficacy Index (TEI) for each indicator at each time point by normalizing the ADSC-treated value to its time-matched AR control, thereby providing a uniform metric across durations. All indicators were oriented so that higher TEI values indicate greater therapeutic benefit. Heatmap and radar chart visualizations confirmed the superior efficacy of 4-week ADSC administration, with the highest number of favorable detection indicators (**Figures 7C, D**). These data collectively demonstrate that among the three treatment cycles, the 4-week ADSC treatment cycle achieved the best therapeutic outcomes in OVA-stimulated AR mice.

## 4 Discussion

In this study, we assessed the anti-allergic effects of systemically delivered ADSCs in AR mice, measuring multiple indicators and comparing treatment cycles. We found that long-cycle (4-weeks) administration achieved superior therapeutic efficacy than short-cycle (1-week and 2-weeks). Clinical AR typically presents with nasal congestion, rhinorrhea, nasal/palatal itch, and sneezing (40, 41). In ovalbumin (OVA)-sensitized mouse models, the numbers of sneezes and nasal rubbing episodes recorded within the first 15 minutes after allergen challenge serve as sensitive quantitative endpoints of AR severity (42, 43). Pathobiologically, several OVA-induced AR studies have shown that eosinophil infiltration and goblet cell hyperplasia are reduced after corticosteroid or immunomodulatory treatment (44–47). In our study, we first examined the behavioral patterns and changes in nasal inflammation to explore the treatment effect of allergic symptoms in OVA-induced AR mice using ADSCs. Then, our results indicate that ADSC administration attenuated AR symptoms, reducing sneezing and nose-rubbing frequencies, and eosinophil and goblet cell infiltration, thereby effectively mitigating OVA-induced nasal inflammation (**Figure 3**). AR induction produced a typical Th2 bias. Serum IgE and IL-4 significantly increased, whereas IFN-γ significantly decreased (48, 49). After ADSC treatment, this Th1/Th2 imbalance was reversed, with serum IgE, IgG1, and TGF-β decreasing (**Figure 4**). While ADSC treatment did not produce a clear elevation in serum IFN-γ, it significantly lowered IL-4 levels, resulting in an increased IFN-γ/IL-4 ratio. These results show that ADSCs can correct the Th1/Th2 skewing seen in allergic responses. Importantly, the IFN-γ/IL-4 ratio consistently decreased after AR induction and increased after ADSC treatment in both ELISA and qRT-PCR, indicating a shift of the overall Th1/Th2 balance toward Th1 polarization (**Figures 4**, **5**).

Evidence indicates that the therapeutic effect of stem cell treatment closely depends on dosing intervals (50). ADSCs and other MSCs exert immunoregulatory and anti-inflammatory actions mainly via paracrine signals rather than long-term engraftment. Prolonging or repeating administration can continuously deliver regulatory signals and gradually rewire the immune network, yielding more durable and consistent outcomes (51–53). Thus, we compared ADSC treatment for 1 week, 2 weeks, and 4 weeks. All regimens alleviated the pathological features of AR in mice. Only the 4-week regimen produced significant improvements in nearly all indicators. The 1-week regimen exhibited improving trends across inflammatory markers and cytokines, with statistical significance observed only for the Th1/Th2 balance. The 2-week regimen was less stable, with few significant changes despite overall improving trends. Variations in the degree of AR induction, immune activation, and responsiveness to ADSC treatment can lead to heterogeneous cytokine and symptom profiles at this intermediate stage. As immune status stabilizes over time, these individual fluctuations may diminish, contributing to the more consistent and pronounced therapeutic effects observed at 4 weeks. Thus, 1 week gave partial benefit, 2 weeks introduced greater interindividual variability, and 4 weeks achieved consistent, optimal effects across symptoms and immune readouts. Overall, the duration of ADSC treatment directly influences the immunomodulatory effects as reported (52). Short-term therapy mainly provides rapid suppression of inflammation, whereas prolonged treatment facilitates the restoration of immune homeostasis, leading to more stable and comprehensive therapeutic outcomes.

In AR models, mesenchymal stem cells (MSCs) can be administered through various routes, including systemic intravenous injection and local nasal or mucosal delivery. As demonstrated previously with ADSC-EVs in our study, the two approaches can be optimized or combined to achieve maximal therapeutic benefit (26, 31). Local administration increases mucosal exposure and tissue concentration while reducing systemic exposure and related risks (54). However, it may be limited by mucociliary clearance, uneven tissue distribution, and challenges in repeated dosing. Compared with local delivery, systemic administration allows the distribution of paracrine signals throughout the body, and influences immune organs such as the spleen and lymph nodes, providing a broader and more integrated immune remodeling effect (29). In this study, we selected tail-vein infusion of ADSCs as a systemic route. Although ADSC-EVs show promise in immune modulation and inflammation control in our prior study (26), they lack the capacities of living cells to sustain metabolism, sense microenvironmental cues, respond to stimuli, and secrete additional factors (55–57). Direct injection of ADSCs may therefore provide advantages in homing, short-term retention, local immune regulation, and tissue repair (58, 59). In this study, injected ADSCs reached the nasal mucosa and remained there for a short period (**Figure 2**), which is consistent with the findings of Cho et al. and colleagues (60). These results support the chemotactic migration of ADSCs to inflamed sites in allergic rhinitis. Other studies also show that MSCs or ADSCs can home to or engraft in injured tissues and then promote repair or immunomodulation (61). Consistent with this, ADSCs have shown immunomodulatory efficacy in AR models. Taken together, evaluating the effects of different administration routes of ADSCs and comparing the therapeutic efficacy between ADSCs and their EVs will be important directions for our future studies, thereby defining optimal strategies for clinical translation.

This study has several limitations that warrant acknowledgment. First, the use of a single mouse cohort and staggered dosing introduced age differences among treatment groups, potentially modifying treatment effects, even though TEI was applied for normalization across time points (62, 63). Besides, we did not conduct an *a priori* power analysis. The group sizes were guided by effect sizes and variability observed in prior studies using this model. This may reduce sensitivity to modest effects and increases the risk of type II error. Furthermore, our immune profiling centered on Th1/Th2 imbalance and associated cytokines without evaluating Th17 cells or regulatory T cells (Tregs). MSC/ADSC products can suppress Th17 responses and promote Tregs through multiple mechanisms (64, 65), MSC-derived small EVs have been demonstrated to restore epithelial barrier integrity (66), highlighting a potentially overlooked mechanism in ADSC immunomodulation. Lastly, clinical translation issues also merit caution. Long-term safety of systemic ADSC therapy was not assessed in the AR model, including safe dosing, immunogenicity, and potential tumorigenicity (67, 68), all of which are prerequisites for clinical translation. Cellular stability and reproducibility are additional constraints. We did not assess ADSC heterogeneity, including donor variability, batch differences, cellular senescence, and passaging drift (69, 70). These factors can alter immunomodulatory capacity and therapeutic consistency, leading to variability in efficacy. In future work, we will concentrate on defining the treatment paradigms and mechanisms of ADSC therapy in the AR mouse model, selecting preclinical strategies with clear translational potential, and systematically addressing the limitations noted above.

## 5 Conclusion

Systemic ADSC delivery mitigated OVA-induced AR by lowering eosinophils and goblet cells, dampening sIgE, IgG1 and TGF-β, and rebalancing Th1/Th2 immunity. Although a 1-week course was effective, the 4-week ADSC treatment cycle achieved the best therapeutic outcomes. These findings supported prolonged, multi-cycle ADSC therapy as a promising approach for clinical AR.

## Data Availability

The original contributions presented in the study are included in the article/supplementary material, further inquiries can be directed to the corresponding authors.
